# Experimental Study and Calculation of a Three-Dimensional Finite Element Model of Infiltration in Drainage Asphalt Pavement

**DOI:** 10.3390/ma13183909

**Published:** 2020-09-04

**Authors:** Qingsong Zhang, Tianjian Ji, Zhanqi Wang, Lei Xiao

**Affiliations:** Department of Civil Engineering, Nanjing University of Aeronautics and Astronautics, Nanjing 210000, China; zqs1019@nuaa.edu.cn (Q.Z.); wangzq911201@nuaa.edu.cn (Z.W.); lei_xiao1995@163.com (L.X.)

**Keywords:** drainage asphalt pavement, three-dimensional infiltration finite element method, single-sided water penetration test, longitudinal slope drainage

## Abstract

Drainage asphalt pavement provides excellent drainage performance and driving safety, where the permeability of the pavement is the critical performance. When analyzing the permeability of drainage asphalt pavements, the previously used two-dimensional infiltration calculation model with boundary conditions deviates from the real situation. In this article, a three-dimensional infiltration finite element method was proposed to evaluate the permeability of pavement, and the feasibility of the three-dimensional infiltration finite element method to evaluate the drainage capacity of drainage asphalt pavement was verified by using the single-sided permeability test of indoor rutted slabs of drainage asphalt mixtures as an example. Finally, the effects of the longitudinal slope of the pavement on the drainage performance of the drained asphalt pavement was investigated by calculations based on the three-dimensional infiltration finite element method. The results indicated that: when the longitudinal slope was less than 6%, the influence of longitudinal slope size on the drainage capacity of drainage asphalt pavement was very small. When the gradient of the longitudinal slope from 0 to 6%, the critical drainage capacity of the pavement corresponding to each cross slope was maintained at a relatively stable value.

## 1. Introduction 

Drainage asphalt pavement, also known as permeable asphalt pavement, refers to a new type of asphalt mixture surface layer that has a porosity of about 15–23% after compaction and can form a drainage channel inside the asphalt mixture [1]. Its essence is a single-grain crushed stone according to the principle of embedding and extrusion of aggregates to form the skeleton of the open-graded asphalt mixture-void structure [2].

Different from the dense structure of the traditional pavement, the drainage asphalt pavement mostly uses large void asphalt mixture as the drainage surface layer [3]. The middle and lower asphalt surfaces of the drainage surface layer are dense asphalt mixture, which can form a drainage channel inside the asphalt mixture. In this way, the water seeping into the drainage layer on rainy days can be discharged out of the pavement structure along the pavement slope [4,5,6]. The drainage characteristics of drainage asphalt pavements are indicated by their permeability: the higher the permeability, the greater the drainage capacity. Thus, fully understanding and effectively determining the permeability of drainage asphalt pavements is the key to successful application of drainage asphalt pavements.

In 1999, the National Center for Asphalt Technology (NCAT) developed an in-situ OGFC (Open Graded Friction Course) permeability testing device based on the variable-head principle, which is now one of two commonly used pavement permeability testing methods in the U.S. It is widely used to evaluate the permeability of permeable asphalt pavements and open-graded permeable thin layers. Khalifa et al. [7] compared these two permeability testing methods and found that both methods can be applied to test the permeability of all pavements and the accuracy of the test results is not affected by the pavement material. Chen et al. [8] tested the coefficient of permeability of OGFC pavements using the Florida State compaction asphalt mixture test method (variable head method). Mohammad et al. [9] found that the water permeability of asphalt pavement is related to the type of asphalt mixture, pavement field porosity, structural layer thickness, and pavement surface structure depth by testing the water permeability coefficient of asphalt pavement in three highways under construction. Masad et al. [10] proposed to use a simple linear equation to approximate the permeability coefficient of asphalt concrete. The equation parameters include mixture porosity and aggregate substitution, and the calculation results of the equation are in good agreement with many field and indoor test results.

(1) $$k=\frac{V_{a}^{m}}{c\cdot S_{agg}^{t}}\frac{\gamma }{\mu }$$

where: γ—The mass of fluid per unit volume, $\mathrm{KN}/m^{3}$, $9.79\;\mathrm{KN}/m^{3}$ at 20 °C water temperature; *μ*—Water viscosity, take 0.001 kg/(m·s). The *c*, *m* and *t* values are obtained through statistical data fitting to the permeability values expressed in the units of $10^{-5}$.

In order to better evaluate the water permeability of pavements, researchers have improved the traditional test apparatus and methods for measuring pavement permeability coefficients. Self-developed permeability coefficient tester was used for the first time to test the permeability coefficients of different void ratios and graded test pieces, and analyzed the factors affecting the permeability of asphalt mixture [11,12]. Zheng [13] analyzed the physical basis of the permeability coefficient, and developed a simple and practical porous concrete permeation instrument based on the principle of the permeability test of the normal head. Ma et al. [14,15] established the relationship among porosity, effective porosity, vertical permeability coefficient, and lateral permeability coefficient through the test results of the improved lateral permeability coefficient tester.

Most current scholars, when performing infiltration process analysis, infiltration line modeling, and drainage capacity calculations for drained asphalt pavements, viewed drained asphalt pavements as a special case where the longitudinal slope was zero [16]. This simplified the three-dimensional infiltration process into a two-dimensional infiltration process, which facilitated the extrapolation of the model and the calculation of the results. Because of the existence of longitudinal slope, lengthening the drainage path, but also increase the head difference, the former slows down the water discharge within the pavement structure, and the latter accelerates the water discharge [17]. How the combined effect of the two affects the drainage performance of the drainage pavement needs further study.

Based on the basic theory of three-dimensional finite element infiltration, this paper established the differential equation for stable infiltration. This model was used to simulate the indoor rutted asphalt mixture single-sided infiltration test. Then results of indoor rutted plate single-sided infiltration test and the infiltration model based on the three-dimensional infiltration finite element method were compared to verify whether the infiltration model can be used to simulate the infiltration process of rainwater in a drained asphalt mixture. For the drainage asphalt pavement infiltration process analysis and the calculation of drainage capacity, this could provide a new way of thinking. The validated infiltration model was used to evaluate the drainage capacity of the drainage asphalt pavement by incorporating it into the calculation of the actual conditions.

## 2. Basic Model of Infiltration in Drainage Asphalt Pavement

### 2.1. Three-Dimensional Finite Element Infiltration Basic Equation

**Figure 1:**
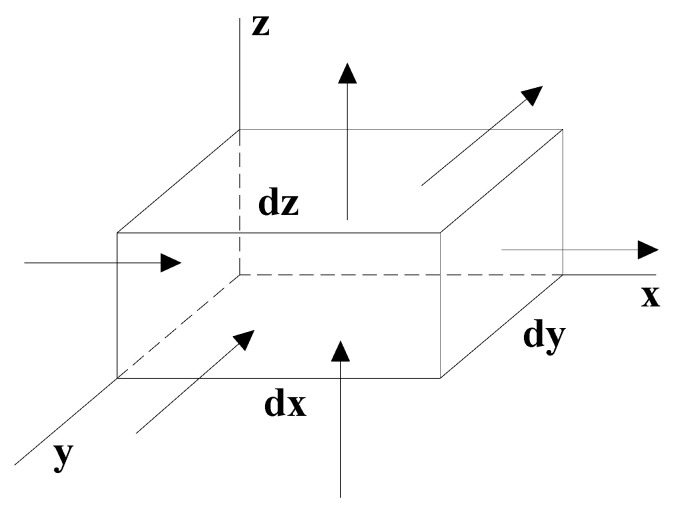
Schematic diagram of inflow and outflow on each face of the differential unit.

The infiltration continuity equation is based on the principle of conservation of mass, while taking into account the compressibility of the medium. That is, the rate of increase and decrease of the infiltration field in any unit is equal to the difference between the flow rate of the unit and the unit. Figure 1 is a schematic diagram of the flow in and out of each face of the finite element differential unit body. Each side of the differential unit body is parallel to the *x*, *y*, and *z* axes, and $d_{x}$, $d_{y}$, and $d_{z}$ are the lengths of the sides of the differential unit body. Suppose that the infiltration velocity components along the *x*, *y*, and *z* axes are $v_{x}$, $v_{y}$, and $v_{z}$, and the fluid density is ρ.

As shown in Figure 1, at a certain instant t, the coordinates of the center point of the unit body are (*x*, *y*, *z*), and the density and velocity are respectively ρ, u.

In the x-direction, for example, by expanding the Taylor series and omitting the second-order and higher items in the series, the velocities and densities of the left and right sides are as follows: $u_{x}-\frac{\partial u_{x}}{\partial x}\frac{dx}{2}$ and $u_{x}+\frac{\partial u_{x}}{\partial x}\frac{dx}{2}$, $\rho -\frac{\partial \rho }{\partial x}\frac{dx}{2}$ and $\rho +\frac{\partial \rho }{\partial x}\frac{dx}{2}$. Since each boundary area of the micro unit body is small, it can be considered that the velocity and density of each point on the same surface are the same. Therefore, in the dt time period, the difference between the mass of the liquid flowing in and out of the unit body in the *x* direction can be expressed as:

(2) $$(\rho +\frac{\partial \rho }{\partial x}\frac{dx}{2})(\;u_{x}+\frac{\partial u_{x}}{\partial x}\frac{dx}{2})dydzdt-(\rho -\frac{\partial \rho }{\partial x}\frac{dx}{2})(\;u_{x}-\frac{\partial u_{x}}{\partial x}\frac{dx}{2})dydzdt\;=\;\frac{\partial \left(\rho u_{x}\right)}{\partial x}dxdydzdt$$

Similarly, the difference between the mass of the liquid flowing in and out of the unit body along the *y* *z* direction is $\frac{\partial \left(\rho u_{y}\right)}{\partial y}dxdydzdt$ and $\frac{\partial \left(\rho u_{z}\right)}{\partial z}dxdydzdt$.

According to the law of conservation of mass, during the dt period, the difference between the mass of the liquid flowing out and in the unit is equal to the change in mass in the unit. which is

(3) $$[\frac{\partial \left(\rho u_{x}\right)}{\partial x}+\frac{\partial \left(\rho u_{y}\right)}{\partial y}+\frac{\partial \left(\rho u_{z}\right)}{\partial z}]\;dxdydzdt=-\frac{\partial }{\partial t}\left(\rho dxdydz\right)dt$$

After simplification, the differential form of the continuity equation in the Cartesian coordinate system is obtained:

(4) $$\frac{\partial \rho }{\partial t}+\frac{\partial \left(\rho u_{x}\right)}{\partial x}+\frac{\partial \left(\rho u_{y}\right)}{\partial y}+\frac{\partial \left(\rho u_{z}\right)}{\partial z}=0$$

For a constant flow,$\;\frac{\partial \rho }{\partial t}=0$, the continuity equation is

(5) $$\frac{\partial \left(\rho u_{x}\right)}{\partial x}+\frac{\partial \left(\rho u_{y}\right)}{\partial y}+\frac{\partial \left(\rho u_{z}\right)}{\partial z}=0$$

If the liquid is an incompressible liquid, the density derivative can be canceled out [18], then Equation (5) can be rewritten as

(6) $$\frac{\partial \left(u_{x}\right)}{\partial x}+\frac{\partial \left(u_{y}\right)}{\partial y}+\frac{\partial \left(u_{z}\right)}{\partial z}=0$$

Equation (6) is the infiltration continuity equation, which shows that at any moment in the unit of fluid mass change rate is zero. The unit of infiltration in a direction to change the direction of infiltration must have other changes in equilibrium with it.

### 2.2. Three-Dimensional Stable Infiltration Differential Equation

When the permeation medium is anisotropic, substituting Darcy’s law into Equation (6), the differential equation for stable infiltration is obtained:

$$v_{x}=-K_{x}\frac{\partial h}{\partial x}v_{y}=-K_{y}\frac{\partial h}{\partial y}v_{z}=-K_{z}\frac{\partial h}{\partial z}$$

(7) $$\frac{\partial }{\partial x}\left(K_{x}\frac{\partial h}{\partial x}\right)+\frac{\partial }{\partial y}\left(K_{y}\frac{\partial h}{\partial y}\right)+\frac{\partial }{\partial z}\left(K_{z}\frac{\partial h}{\partial z}\right)=0$$

where: *h*—total water head;

*K_x_*—The permeability coefficient in the *x*-axis direction;

*K_y_*—The permeability coefficient in the *y*-axis direction;

*K_z_*—he permeability coefficient in the *z*-axis direction.

When the permeability coefficient of the permeable medium does not change with time, Equation (7) becomes:

(8) $$K_{x}\frac{\partial^{2}h}{\partial x^{2}}+K_{y}\frac{\partial^{2}h}{\partial y^{2}}+K_{z}\frac{\partial^{2}h}{\partial z^{2}}=0$$

When the permeation medium is isotropic, Equation (8) becomes the Laplace equation:

(9) $$\frac{\partial^{2}h}{\partial x^{2}}+\frac{\partial^{2}h}{\partial y^{2}}+\frac{\partial^{2}h}{\partial z^{2}}=0$$

Equation (9) is the differential equation for stable infiltration, but the instantaneous stability field can also be calculated for incompressible media and fluids with unstable flow. Since the Laplace equation for percolation has only one unknown, it can be solved by combining it with known boundary conditions. The mathematical model used in the 3D infiltration finite element numerical analysis software used in this paper is derived from the Laplace equation and solved according to the specific head boundary conditions and flow boundary conditions.

## 3. Numerical Simulation and Experimental Validation of Percolation in Drainage Mixtures

### 3.1. Numerical Simulation of Percolation in Drainage Mixtures

With the development of computer numerical simulation technology, the finite element analysis (FEA) method, with its many advantages, has become an important analysis method for solving engineering problems. In this section, the three-dimensional infiltration finite element method was used to investigate the infiltration model of rutted slabs under single-sided infiltration conditions and compare it with the experimental results to verify the feasibility of the three-dimensional infiltration finite element method.

#### 3.1.1. Three-Dimensional Finite Element Infiltration Simulation for Single-Sided Water Infiltration Test of Rutted Slabs

Since the use of three-dimensional infiltration finite element software to calculate drainage asphalt pavement drainage performance lacked the corresponding application experience, this paper first validated the effectiveness of the finite element numerical simulation. In combination with the indoor rutted slab single-sided water infiltration test specific cases, according to the actual size of the rutted slab thickness of 10 cm to build a model, and calculated the infiltration of the drainage pavement under different head heights. The main steps of the simulation calculation were as follows:

(1) Define the infiltration pattern

The percolation velocity of rainwater within the drained asphalt pavement is low, and the percolation process is close to the steady flow state, which was set to steady percolation and nonlinear superposition in this case.

(2) Develop the finite element model

Using COMSOL Multiphysics as analysis software. The indoor single-sided infiltration test with drainage asphalt mixture rutting plate specimens’ size of 30 cm × 30 cm × 10 cm and the size of the finite element model was the same as the size of the rutted plate specimen.

(3) Divide the grid of the finite element model

**Figure 2:**
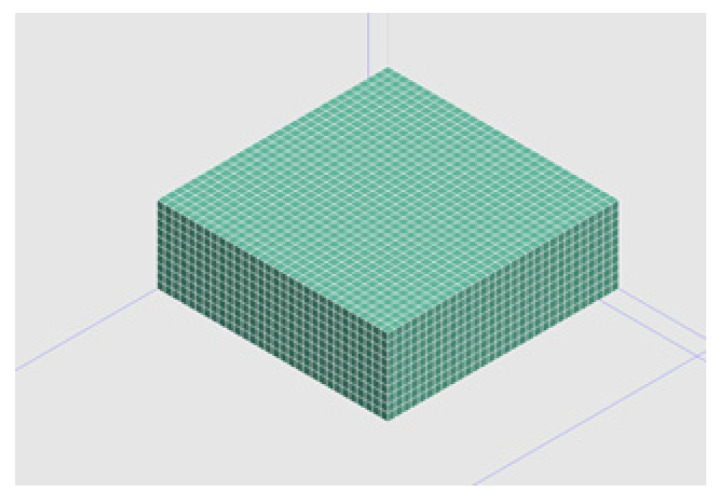
Geometric model of geometry for the calculation of infiltration on a drainage asphalt pavement.

The division of the finite element model of the 3D infiltration finite element calculation software was square as appropriate. The size of the finite element model was 30 cm × 30 cm × 10 cm, where the model was divided into 9000 units, each grid cell was a cube with 1 cm side length. Through several debugging efforts, it was found that the grid division can meet the accuracy requirements, and the solution time was reasonable. The grid division effect is shown in Figure 2.

(4) Establish boundary conditions

**Figure 3:**
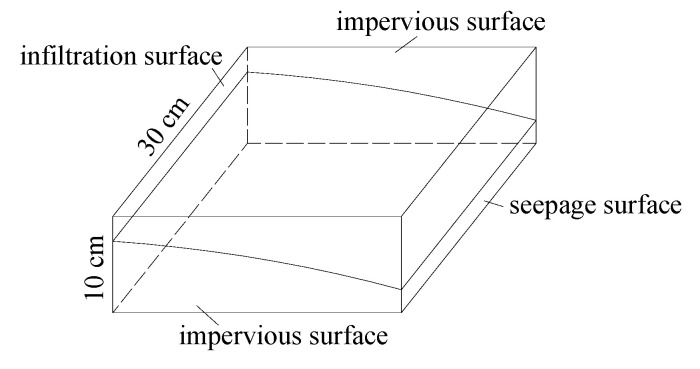
Schematic diagram of boundary conditions.

This three-dimensional infiltration finite element software simulated the indoor one-sided water storage test of the drainage asphalt mixture rut. The infiltration model can be built with three boundary conditions:

① The contact part between the test piece of the rut plate and the test mold was an impervious surface;
② Free infiltration surface, including water infiltration surface and exudation surface;
③ The boundary condition of the head was equal to the height of the head corresponding to the infiltration surface and the outflow surface of the laboratory test. The schematic diagram of the boundary condition setting is shown in Figure 3.

(5) Material permeability coefficient setting
(6) The material properties section supports setting the permeability coefficient for each direction of the material. Since the drainage asphalt mixture was nearly isotropic, the permeability coefficients were set as the same value in all directions and the value was taken from the test measured permeability coefficient.Result solution

**Figure 4:**
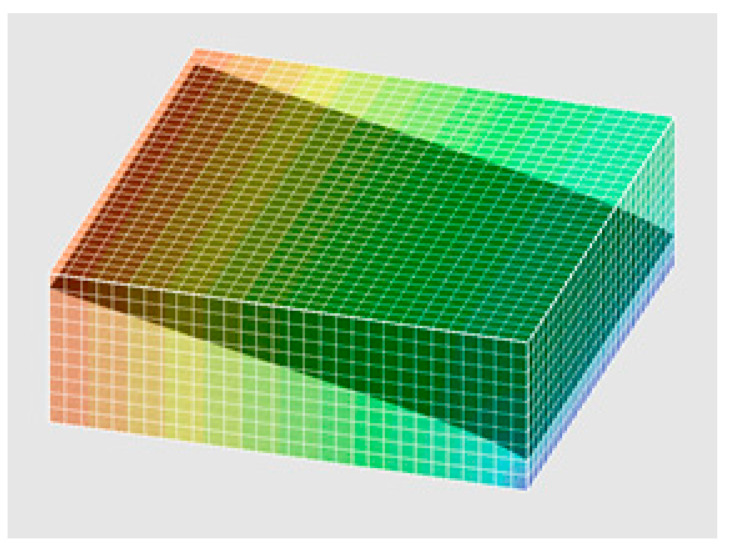
Pressure head cloud diagram for a single-sided infiltration model of drainage asphalt material rutted slabs.

After setting and saving the infiltration mode, including boundary conditions and material parameters, the 3D finite element infiltration simulation can be carried out. The pressure head cloud diagram of the calculation result in one case is shown in Figure 4, and the infiltration flow of the infiltration surface can be found in the calculation result.

#### 3.1.2. Numerical Simulation of Rutted Plate Model

Following the steps in (1)–(6) above to simulate and analyze the infiltration of PAC16 (Porous Asphalt Concrete) rutted plate specimens with different porosity at different head heights, the porosity of the rutting plate specimens of the permeability coefficient of indoor test results are shown in Table 1. In this test, the PAC16 rut board was taken as an example. The results of the finite element numerical simulation of the flow rate per unit area of the test piece at the height of the test head are shown in Table 2. The results of the finite element numerical simulation of the test head height corresponding to the height of the infiltration flow are shown in Table 3.

### 3.2. Experimental Testing and Analytical Validation of the Rutted Slab Infiltration Model

#### 3.2.1. Measurement Method and Test Equipment of Permeability Coefficient

**Figure 5:**
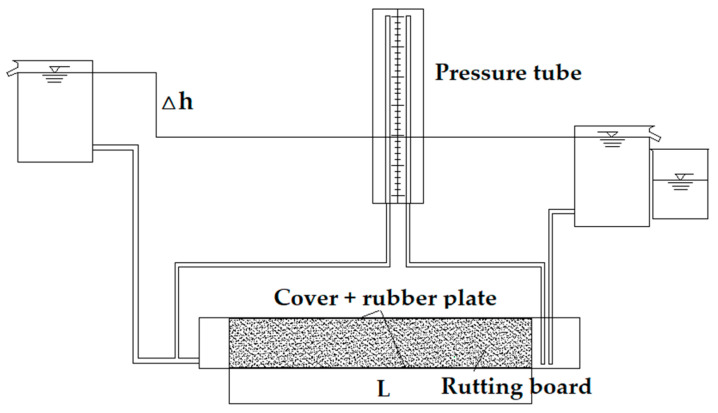
Transverse permeability tester for rutted slab.

In the analysis of the permeation pattern of PAC16 rutted plate specimens, the transverse permeation system of the rutted plate needed to be measured. This study drew on the design experience of the Marshall Vertical Permeability Coefficient Tester based on the principle of constant water head, and designed a lateral permeability coefficient tester for rut boards based on the principle of constant water head. The schematic diagram is shown in Figure 5.

During the test, the formed rut test piece was wrapped with pearl wool, and the infiltration surface and the outflow surface did not need to be wrapped. Then the test piece was placed in the sink and the iron sealing surface was tightly sealed. Finally, the infiltration test can be carried out according to the steps of the principle of constant head. It was found that the pearl cotton has good impermeability, which can ensure the accuracy of the test results.

#### 3.2.2. Test Process and Results

**Figure 6:**
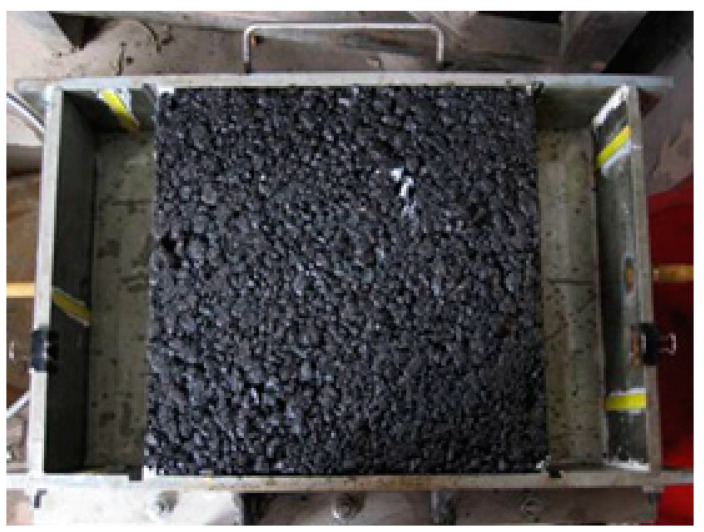
Single-sided infiltration test device for rutted slab.

In this study, the unilateral infiltration model test of the drainage asphalt mixture rut specimen was used to verify the calculation results of the three-dimensional infiltration finite element method. Figure 6 shows an indoor test device with an adjustable left sink height and a 1.5 cm outlet height for the right sink. The test was conducted by continuously filling the left sink with water to maintain a constant water level in the left sink. After the infiltration process has stabilized, the height of the water level in the right sink and the flow rate at the infiltration surface over a certain period of time were measured. Table 4 shows the flow rate at the infiltration surface of the PAC16 rutting plate specimens with different porosity at different head heights. Table 5 shows the measured head heights of the infiltration surface corresponding to different head heights on the infiltration surface.

The analysis of Table 3 and Table 5 shows that the head height at the infiltration surface is very similar to the calculated results of the three-dimensional infiltration finite element numerical simulation for the single-sided infiltration model test at a certain infiltration surface head height. However, the measured head height at the infiltration surface is slightly higher than the finite element numerical simulation method, which can be used as an approximation of the head height at the infiltration surface due to the influence of the control outlet height. Then, the head height at the infiltration surface derived from the three-dimensional finite element numerical simulation was used as the control head, and the parameters of the test model were substituted into the formula of the theoretical model of infiltration, and the calculated flow value can also be obtained. In order to facilitate the comparison of the results obtained by the theoretical method and the finite element simulation method, the flow unit was unified to E-005m3/s, and the detailed results are shown in Table 6.

As can be seen from the results in Table 4 and Table 6, the infiltration flow values calculated according to the theoretical formula derived from the one-dimensional asymptotic flow theory [19] are significantly different from the flow values calculated from the indoor rutted slab infiltration test and the 3D infiltration finite element simulation, which are much smaller than the values of both. This is because the assumptions of the one-dimensional gradual flow theoretical model were quite different from the boundary conditions of the rutted plate unilateral infiltration test. Infiltration was no longer gradual flow near the infiltration surface, which has changed from gradual flow to rapid flow, so the actual value of the cross-sectional infiltration flow should be greater. The theoretical model was used for infiltration calculations of drained asphalt mixtures, and the assumptions of the model should be as consistent as possible with the actual infiltration conditions, otherwise the calculation result will seriously deviate from the true value.

From the data in the table, it can also be found that the measured flow rate results of the test are closer to the results of the FEM simulation, and slightly smaller than the results of the numerical calculation, and the numerically calculated head height at the infiltration surface is significantly smaller than the test case. The main reason for this is that the outlet set up in the indoor test will cause a congestion phenomenon at the outlet, which indirectly causes the infiltration flow measured in the test to be smaller than the calculated value of 3D infiltration finite element simulation. This shows that the boundary conditions of the finite element numerical simulation model are more reasonable and closer to the actual infiltration of the drainage layer. The use of three-dimensional infiltration finite element analysis software to simulate drainage asphalt pavement infiltration conditions can not only visually reflect the internal infiltration of the drainage layer infiltration surface, but also can view the flow of any section of the condition. This is feasible for the analysis of the drainage capacity of drainage asphalt pavement, and it is also very convenient.

## 4. The Effect of Longitudinal Slope on Drainage Performance of Drained Asphalt Pavements

During the design of road cross-section at all levels, a lateral slope of 1.5 to 2% is required in order to facilitate the timely drainage of rainfall from the road surface [20]. In the process of road longitudinal section design, in order to facilitate the longitudinal drainage of the side ditch and the consideration based on terrain, the general design is a minimum longitudinal slope of 0.5%. The maximum longitudinal slope is determined by road design speed, terrain, and other conditions. The Road Route Design Code stipulates that the design speed is 120 km/h, 100 km/h, 80 km/h, 60 km/h, and the corresponding maximum design longitudinal slope is 3, 4, 5, and 6% [21].

While the longitudinal slope of the road changes, the synthetic slope of the road surface and the length of the drainage path also change [22]. In the traditional two-dimensional infiltration calculation process, the drainage asphalt pavement was regarded as a special case where the longitudinal slope was zero, and the influence of the longitudinal slope on the infiltration process was ignored. The contradiction between the influence of the combined slope of the pavement and the drainage length on the drainage performance of the drainage asphalt pavement due to the longitudinal slope of the pavement design is inevitable [23,24,25]. Therefore, to find out the influence law of the longitudinal slope of the road on the drainage capacity for the design of the drainage asphalt pavement is very helpful. In order to analyze the influence of longitudinal slope on the critical drainage strength of drainage asphalt pavement, this section used a pavement width of 10 m (drainage length 5 m), a length of 80 m, and a drainage layer thickness of 5 cm, and a common engineering value as the road calculation model. The critical drainage strength of the pavement corresponding to different longitudinal slopes was calculated using the 3D infiltration finite element method.

### 4.1. Two Boundary Conditions in the Presence of Longitudinal Slopes

**Figure 7:**
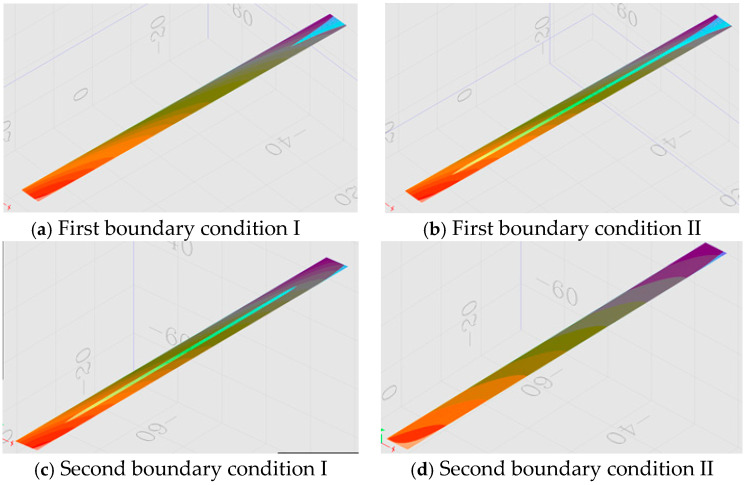
Head cloud diagram with two boundary conditions.

When there are longitudinal slopes, the infiltration process of rainwater in the drainage layer becomes more complex. In order to make this simulation as close as possible to the actual rainfall infiltration process, two boundary conditions were set up [26,27,28]. The first was where the boundary conditions on both longitudinal sides of the road were set at impervious surfaces, i.e., where no incoming or outgoing flows occur, in which case the incoming and outgoing flows were closer to the actual conditions and the second was that the higher longitudinally positioned sides of the road were set up as impervious and the lower positioned sides were set up as pervious, which was more similar to the actual infiltration condition of the pavement. The first boundary condition head cloud diagram is shown in Figure 7a, where the road surface is experiencing runoff, and Figure 7b, where the infiltrated surface is tangent to the road surface in the middle of the road where no runoff occurs. Figure 7c, where the second boundary condition head cloud diagram is shown, where the road surface is experiencing runoff, and Figure 7d, where the road surface is experiencing runoff in the middle of the road where no runoff occurs.

### 4.2. Calculation Results Based on 3D Infiltration Finite Element Method

Since the synthetic slope is the result of the combined effect of the transverse and longitudinal slopes of the road [29,30,31,32], the critical drainage strength of the pavement was calculated for the transverse slope of 1 and 4% corresponding to different longitudinal slopes (0–6%), respectively. The permeability coefficients K (cm/s) were 0.3, 0.6, 0.9, 1.2, and 1.5, respectively. The boundary conditions for both cases were also considered, and some of the calculations are listed in Table 7, Table 8, Table 9 and Table 10.

By analyzing the calculation results in Table 7 and Table 8, it can be found that the critical drainage capacity of the pavement increases simultaneously through the increase in the permeability coefficient K of the pavement material. At the same time, under this calculation model, when the slope of the longitudinal slope increases from 0 to 6%, the critical drainage capacity of the road surface corresponding to each cross slope remains at a relatively stable value.

Table 7 and Table 9, Table 8 and Table 10 are the calculation results of the drainage capacity of the pavement with two different boundary conditions. According to the data in the table, although the boundary conditions are different, the calculated critical drainage strength is the same. It can be seen that the setting of the boundary conditions has a greater impact on the pavement penetration within a small range on both sides, but basically has no effect on the drainage capacity of the entire pavement, which is also obvious from the head cloud diagram.

In this article, the effect of longitudinal slope of pavement on drainage performance was analyzed using an experimentally validated three-dimensional infiltration finite element model. Subsequent studies can address the effects of cross slope, drainage layer thickness, drainage length, and surface material permeability coefficient on the drainage capacity of the pavement. The finite element model based on three-dimensional seepage will be used to evaluate the drainage capacity of the road with different pavement widths and different combinations of longitudinal and transverse slopes.

In addition, the internal porosity, pore size, pore curvature, and pore shape of the asphalt mixture have an impact on drainage. This is the direction the research team is working to tackle, and further research is needed.

## 5. Conclusions

1. The rut board lateral permeability coefficient tester based on the principle of normal head test had good sealing performance, which was convenient for indoor measurement of the permeability coefficient of drainage asphalt mixture, and the test results were stable and reliable. It was very suitable for accurately measuring the lateral permeability coefficient of drainage asphalt mixture.
2. Through experiments and calculation verification, it was found that the flow value derived from the infiltration flow formula based on the one-dimensional infiltration principle was obviously small. Therefore, the setting of boundary conditions needed to be optimized. The three-dimensional infiltration finite element method can be used to simulate the rainwater infiltration process inside the drainage asphalt pavement, and this method can be used to calculate and analyze the drainage capacity of the drainage asphalt pavement.
3. The critical drainage capacity of the pavement was directly proportional to the permeability coefficient of the asphalt mixture in the drainage layer.
4. When the longitudinal slope is less than 6%, the effect of the longitudinal slope size on the drainage capacity of the drainage asphalt pavement was very small. When the slope of the longitudinal slope increased from 0 to 6%, the critical drainage capacity of the road surface corresponding to each cross slope remained at a relatively stable value. When the longitudinal slope was 0 to 6%, the combined slope size and drainage path length increased together, and the impact of the two on the drainage capacity of the drainage asphalt pavement was basically cancelled. At this time, the impact of the longitudinal slope on the drainage capacity of the road surface can basically be ignored.

## Figures and Tables

**Table 1:** Permeability coefficients of PAC16 rut test specimens with different void content.

Porosity (%)	Permeability Coefficient (cm/s)
15	0.48
18	0.93
21	1.39
23	1.68

**Table 2:** Three-dimensional finite element calculation results of infiltration flow corresponding to different infiltration surface head heights.

Head of Infiltration Surface Height (cm)	Flow from the Infiltration Surface (m^3^/s)			
	15%	18%	21%	23%
5.0	1.52$\;\times \;10^{-5}$	2.95$\;\times \;10^{-5}$	4.40$\;\times \;10^{-5}$	5.32$\;\times \;10^{-5}$
6.0	1.87$\;\times \;10^{-5}$	3.62$\;\times \;10^{-5}$	5.42$\;\times \;10^{-5}$	6.55$\;\times \;10^{-5}$
7.0	2.22$\;\;\times \;10^{-5}$	4.30$\;\times \;10^{-5}$	6.43$\;\times \;10^{-5}$	7.77$\;\times \;10^{-5}$
8.0	2.59$\;\times \;10^{-5}$	5.02$\;\times \;10^{-5}$	7.50$\;\times \;10^{-5}$	9.07$\;\times \;10^{-5}$
9.0	2.98$\;\times \;10^{-5}$	5.77$\;\times \;10^{-5}$	8.63$\;\times \;10^{-5}$	1.04$\;\times \;10^{-4}$
10.0	3.37$\;\times \;10^{-5}$	6.53$\;\times \;10^{-5}$	9.76$\;\times \;10^{-5}$	1.18$\;\times \;10^{-4}$

**Table 3:** Three-dimensional finite element calculation results of different infiltration surface head heights corresponding to outflow surface head heights.

Infiltration Surface Height (cm)	The Height of the Head of the Infiltration Surface (cm)			
	15%	18%	21%	23%
5.0	1.45	1.45	1.45	1.45
6.0	1.55	1.55	1.55	1.55
7.0	1.65	1.65	1.65	1.65
8.0	1.75	1.75	1.75	1.75
9.0	1.85	1.85	1.85	1.85
10.0	1.95	1.95	1.95	1.95

**Table 4:** Results of infiltration flow test measurements at infiltration surfaces corresponding to different infiltration head heights.

Head Height of Infiltration Surface (cm)	Flow from the Infiltration Surface (m^3^/s)			
	15%	18%	21%	23%
5.0	1.34$\;\times \;10^{-5}$	2.65$\;\times \;10^{-5}$	3.98$\;\times \;10^{-5}$	4.86$\;\times \;10^{-5}$
6.0	1.56$\;\times \;10^{-5}$	3.28$\;\times \;10^{-5}$	5.04$\;\times \;10^{-5}$	6.01$\;\times \;10^{-5}$
7.0	2.01$\;\times \;10^{-5}$	4.01$\;\times \;10^{-5}$	6.01$\;\times \;10^{-5}$	7.16$\;\times \;10^{-5}$
8.0	2.30$\;\times \;10^{-5}$	4.68$\;\times \;10^{-5}$	7.05$\;\times \;10^{-5}$	8.54$\;\times \;10^{-5}$
9.0	2.58$\;\times \;10^{-5}$	5.31$\;\times \;10^{-5}$	8.15$\;\times \;10^{-5}$	9.8$\;\times \;10^{-4}$
10.0	3.02$\;\times \;10^{-5}$	6.21$\;\times \;10^{-5}$	9.12$\;\times \;10^{-5}$	1.12$\;\times \;10^{-4}$

**Table 5:** Test results of head heights corresponding to different infiltration faces.

Head Height of Infiltration Surface (cm)	Height of Water Head of Infiltration Surface (cm)			
	15%	18%	21%	23%
5.0	1.65	1.65	1.65	1.70
6.0	1.75	1.75	1.75	1.78
7.0	1.78	1.80	1.81	1.83
8.0	18.5	1.85	1.87	1.89
9.0	1.90	1.95	1.95	1.98
10.0	2.00	2.03	2.05	2.05

**Table 6:** Comparison table of three-dimensional finite element and numerical theoretical calculation results of infiltration flow at different head heights.

Head Height of Infiltration Surface (cm)	Flow from the Infiltration Surface (10^−5^ m^3^/s)							
	15%		17%		20%		23%	
	AV	TV	AV	TV	AV	TV	AV	TV
5.0	1.34	0.65	2.65	1.26	3.98	1.88	4.86	2.28
6.0	1.56	0.90	3.28	1.74	5.04	2.61	6.01	3.15
7.0	2.01	1.12	4.01	2.17	6.01	3.24	7.16	3.92
8.0	2.30	1.52	4.68	2.95	7.05	4.40	8.54	5.32
9.0	2.58	1.83	5.31	3.55	8.15	5.30	9.8	6.41
10.0	3.02	2.19	6.21	4.24	9.12	6.34	1.12	7.67

Where: AV—Analog value; TV—Theoretical value.

**Table 7:** Calculation result of critical drainage capacity of pavement with different transverse slope of 1% for the first boundary condition.

Longitudinal Slope (%)	Pavement Critical Drainage Capacity (m/s)				
	K1 = 0.3 cm/s	K2 = 0.6 cm/s	K3 = 0.9 cm/s	K4 = 1.2 cm/s	K5 = 1.5 cm/s
0	1.11$\;\times \;10^{-6}$	2.22$\;\times \;10^{-6}$	3.33$\;\times \;10^{-6}$	4.44$\;\times \;10^{-6}$	5.55$\;\times \;10^{-6}$
1	1.11$\;\times \;10^{-6}$	2.22$\;\times \;10^{-6}$	3.33$\;\times \;10^{-6}$	4.44$\;\times \;10^{-6}$	5.55$\;\times \;10^{-6}$
2	1.11$\;\times \;10^{-6}$	2.22$\;\times \;10^{-6}$	3.33$\;\times \;10^{-6}$	4.44$\;\times \;10^{-6}$	5.55$\;\times \;10^{-6}$
3	1.11$\;\times \;10^{-6}$	2.22$\;\times \;10^{-6}$	3.33$\;\times \;10^{-6}$	4.44$\;\times \;10^{-6}$	5.55$\;\times \;10^{-6}$
4	1.11$\;\times \;10^{-6}$	2.22$\;\times \;10^{-6}$	3.32$\;\times \;10^{-6}$	4.43$\;\times \;10^{-6}$	5.54$\;\times \;10^{-6}$
5	1.11$\;\times \;10^{-6}$	2.21$\;\times \;10^{-6}$	3.32$\;\times \;10^{-6}$	4.43$\;\times \;10^{-6}$	5.53$\;\times \;10^{-6}$
6	1.11$\;\times \;10^{-6}$	2.21$\;\times \;10^{-6}$	3.32$\;\times \;10^{-6}$	4.43$\;\times \;10^{-6}$	5.54$\;\times \;10^{-6}$

**Table 8:** Calculation result of the critical drainage capacity of the pavement with different transverse slopes of 4% for the first boundary condition.

Longitudinal Slope (%)	Pavement Critical Drainage Capacity (m/s)				
	K1 = 0.3 cm/s	K2 = 0.6 cm/s	K3 = 0.9 cm/s	K4 = 1.2 cm/s	K5 = 1.5 cm/s
0	2.37$\;\times \;10^{-6}$	4.75$\;\times \;10^{-6}$	7.11$\;\times \;10^{-6}$	9.48$\;\times \;10^{-6}$	11.85$\;\times \;10^{-6}$
1	2.37$\;\times \;10^{-6}$	4.75$\;\times \;10^{-6}$	7.11$\;\times \;10^{-6}$	9.48$\;\times \;10^{-6}$	11.85$\;\times \;10^{-6}$
2	2.37$\;\times \;10^{-6}$	4.75$\;\times \;10^{-6}$	7.11$\;\times \;10^{-6}$	9.48$\;\times \;10^{-6}$	11.85$\;\times \;10^{-6}$
3	2.37$\;\times \;10^{-6}$	4.75$\;\times \;10^{-6}$	7.11$\;\times \;10^{-6}$	9.48$\;\times \;10^{-6}$	11.85$\;\times \;10^{-6}$
4	2.37$\;\times \;10^{-6}$	4.75$\;\times \;10^{-6}$	7.10$\;\times \;10^{-6}$	9.48$\;\times \;10^{-6}$	11.85$\;\times \;10^{-6}$
5	2.37$\;\times \;10^{-6}$	4.74$\;\times \;10^{-6}$	7.10$\;\times \;10^{-6}$	9.47$\;\times \;10^{-6}$	11.84$\;\times \;10^{-6}$
6	2.37$\;\times \;10^{-6}$	4.74$\;\times \;10^{-6}$	7.10$\;\times \;10^{-6}$	9.47$\;\times \;10^{-6}$	11.84$\;\times \;10^{-6}$

**Table 9:** Calculation result of the critical drainage capacity of pavement with different transverse slope of 1% for the second boundary condition.

Longitudinal Slope (%)	Pavement Critical Drainage Capacity (m/s)				
	K1 = 0.3 cm/s	K2 = 0.6 cm/s	K3 = 0.9 cm/s	K4 = 1.2 cm/s	K5 = 1.5 cm/s
0	1.11$\;\times \;10^{-6}$	2.22$\;\times \;10^{-6}$	3.33$\;\times \;10^{-6}$	4.44$\;\times \;10^{-6}$	5.55$\;\times \;10^{-6}$
1	1.11$\;\times \;10^{-6}$	2.22$\;\times \;10^{-6}$	3.33$\;\times \;10^{-6}$	4.44$\;\times \;10^{-6}$	5.55$\;\times \;10^{-6}$
2	1.11$\;\times \;10^{-6}$	2.22$\;\times \;10^{-6}$	3.33$\;\times \;10^{-6}$	4.44$\;\times \;10^{-6}$	5.55$\;\times \;10^{-6}$
3	1.11$\;\times \;10^{-6}$	2.22$\;\times \;10^{-6}$	3.33$\;\times \;10^{-6}$	4.44$\;\times \;10^{-6}$	5.55$\;\times \;10^{-6}$
4	1.11$\;\times \;10^{-6}$	2.21$\;\times \;10^{-6}$	3.32$\;\times \;10^{-6}$	4.43$\;\times \;10^{-6}$	5.55$\;\times \;10^{-6}$
5	1.11$\;\times \;10^{-6}$	2.21$\;\times \;10^{-6}$	3.32$\;\times \;10^{-6}$	4.43$\;\times \;10^{-6}$	5.54$\;\times \;10^{-6}$
6	1.11$\;\times \;10^{-6}$	2.21$\;\times \;10^{-6}$	3.32$\;\times \;10^{-6}$	4.43$\;\times \;10^{-6}$	5.53$\;\times \;10^{-6}$

**Table 10:** Calculation result of the critical drainage capacity of the pavement with different transverse slope of 4% for the second boundary condition.

Longitudinal Slope (%)	Pavement Critical Drainage Capacity (m/s)				
	K1 = 0.3 cm/s	K2 = 0.6 cm/s	K3 = 0.9 cm/s	K4 = 1.2 cm/s	K5 = 1.5 cm/s
0	2.37$\;\times \;10^{-6}$	4.75$\;\times \;10^{-6}$	7.11$\;\times \;10^{-6}$	9.48$\;\times \;10^{-6}$	11.85$\;\times \;10^{-6}$
1	2.37$\;\times \;10^{-6}$	4.75$\;\times \;10^{-6}$	7.11$\;\times \;10^{-6}$	9.48$\;\times \;10^{-6}$	11.85$\;\times \;10^{-6}$
2	2.37$\;\times \;10^{-6}$	4.75$\;\times \;10^{-6}$	7.11$\;\times \;10^{-6}$	9.48$\;\times \;10^{-6}$	11.85$\;\times \;10^{-6}$
3	2.37$\;\times \;10^{-6}$	4.75$\;\times \;10^{-6}$	7.11$\;\times \;10^{-6}$	9.48$\;\times \;10^{-6}$	11.85$\;\times \;10^{-6}$
4	2.37$\;\times \;10^{-6}$	4.74$\;\times \;10^{-6}$	7.11$\;\times \;10^{-6}$	9.48$\;\times \;10^{-6}$	11.85$\;\times \;10^{-6}$
5	2.37$\;\times \;10^{-6}$	4.74$\;\times \;10^{-6}$	7.10$\;\times \;10^{-6}$	9.47$\;\times \;10^{-6}$	11.85$\;\times \;10^{-6}$
6	2.37$\;\times \;10^{-6}$	4.74$\;\times \;10^{-6}$	7.10$\;\times \;10^{-6}$	9.47$\;\times \;10^{-6}$	11.84$\;\times \;10^{-6}$
